# Next generation performance status: digital health technologies across the lung cancer continuum

**DOI:** 10.3389/fdgth.2025.1558180

**Published:** 2025-07-10

**Authors:** Julia Scarpa, Iqram Hussain, Alex Cheng, Jonathan Villena-Vargas, Richard Boyer

**Affiliations:** ^1^ Department of Anesthesiology, Weill Cornell Medicine, New York, NY, United States; ^2^ Weill Cornell Medical College, Weill Cornell Medicine, New York, NY, United States; ^3^ Department of Cardiothoracic Surgery, Weill Cornell Medicine, New York, NY, United States

**Keywords:** functional capacity, performance status, cardiopulmonary health, lung cancer, digital health, wearables

## Abstract

Lung cancer survival rates have greatly benefited from recent advances in therapies and screening. New digital health technologies offer clinicians another way to personalize and enhance care for these patients. For example, emerging technologies support continuous assessment of patients' functional capacity and provide real-time health feedback, improving management of chronic symptoms and monitoring of health trajectory. This review explores these advancements and their potential applications across the lung cancer continuum.

## Introduction

Survival rates in lung cancer have dramatically improved in recent decades following advances in therapeutics and screening technologies. The care of patients across the lung cancer continuum is further elevated by personalized treatment informed by patients' performance status, a strong, independent prognostic factor. Performance status, or functional capacity, describes a patient's capacity to complete activities associated with daily living. The gold standard index of performance status is cardiorespiratory fitness, historically impractical to measure in a clinical setting due to extensive resource requirements. However, recent expansions in personal digital health technologies offer an accessible way for clinicians to assess these factors. With wide-spread access to these data from personal health devices, clinicians are able to continuously monitor their patients, which can be crucial in the management of chronic disease. Additionally, the emergence of artificial intelligence (AI) and machine learning (ML) models has led to real-time health feedback, alerting patients and their providers to potential health complications. This review focuses on these recent advancements in personal device biometric data collection and offers potential applications of these technologies in the treatment of lung cancer. We also present ways in which emerging technologies can supersede the gold-standard prognostic factors via continuous data monitoring and AI-integrated digital health.

## Background

Lung cancer is the most common cancer worldwide with over 2.1 million new cases reported and 1.8 million cancer-related deaths in 2018 (1). The two primary subtypes of lung cancer, non-small cell lung cancer (NSCLC) and small-cell lung cancer (SCLC), represent 76% and 13% of all lung cancers in the United States, respectively (2). Overall mortality from lung cancer is declining, driven by a decreased incidence and improving survival with the introduction of new targeted therapies and immunotherapies (2). Improved lung cancer screening and advanced imaging has also led to an increase in early stage diagnosis and disease amenable to surgical resection, which also likely contribute to improvements in overall survival (3). As lung cancer survival increases and new therapies become available, it is important to identify prognostic factors and phenotypes that can be used to inform treatment planning and chronic disease management across the lung cancer continuum.

Performance status is a strong, independent prognostic factor of overall survival in lung cancer (4). Performance status, also referred to as functional capacity, is a measure of the ability to participate in activities of daily living and is an important determinant of overall health and well-being (5). Historically, performance status was initially defined by Karnofsky in the context of patient responses to lung cancer chemotherapy with nitrogen mustard, but it is now discussed in nearly all chronic medical conditions as an independent predictor of response to therapy and survival. The Karnofsky Performance Status (KPS) scale ranks performance on a scale from 0 to 100, with 0 indicating death and 100 indicating health with no symptoms or evidence of disease. KPS and the closely related Eastern Cooperative Oncology Group (ECOG) score continue to be widely used today for prognostication and risk stratification, although both scales have been criticized because of their subjectivity and low interobserver agreement (6). The gold standard index of performance status—cardiorespiratory fitness (CRF), is an objective and reproducible measure of the ability of the cardiovascular and respiratory systems to supply oxygen during sustained physical activity. Unfortunately, the sophisticated metabolic and physiological monitoring equipment needed for CRF evaluation makes it impractical for most clinical applications, which has prompted clinicians and researchers to search for alternative strategies for performance status assessment and prognostication.

Advancing digital health technologies, including multimodal wearables and consumer health devices, offer an objective, continuous and widely accessible alternative to patient-reported outcomes and formal laboratory assessments of performance status. While early wearable fitness trackers integrated simple triaxial accelerometers to gauge step counts and total activity levels, these devices lacked the resolution and sensors necessary to accurately classify and quantify patient activity. Current consumer wearable devices, such as Fitbit and Apple Watch, integrate advanced analytics with high-performance MEMS triaxial accelerometers, gyroscopes and altimeters for improved tracking of patient activity and estimated energy expenditure (7). In a study of adult patients with cancer receiving systemic therapy, Gupta et al. found that steps per day measured by a wearable activity monitor (*Fitbit Flex*) accurately correlated with clinician-assessed ECOG performance status (ECOG PS) (8). Gresham et al. also evaluated wearable activity monitors (*Fitbit Charge HR*) in 37 advanced cancer patients, and found that average daily steps were correlated to ECOG PS and KPS and each 1,000 steps/day increase was associated with reduced odds for adverse events, hospitalizations and death (9). Although these studies suggest that step counts and activity monitoring can add objectivity to performance status estimates, pedometry alone cannot distinguish between patient effort and exercise intolerance.

Many modern wearable fitness trackers also contain embedded biometric sensors, such as optical sensors for continuous heart rate monitoring and vibration sensors for sleep quality assessment. Integration of accelerometry and biometric sensing in multimodal wearables allows for concurrent assessment of physical activity and physiological response, which creates enormous opportunity for development of advanced models of energy expenditure and performance status (10). For instance, Weyand et al. found that foot-ground contact times and heart rate during ambulating accurately predicted maximal aerobic power, as measured by VO2max using a simple linear regression model (11). More recently, Bonomi et al. used a triaxial accelerometer and chest-belt heart rate monitor to estimate total energy expenditure and VO2max in free-living young adults (12). Few studies have been performed on the use of multimodal wearables for estimates of performance status in cancer. However, a recent study of 41 patients with solid tumor undergoing chemotherapy found that higher average metabolic equivalents (METs) calculated from estimated energy expenditure in a heart rate and activity-tracking wristband (*Microsoft Band 2*) was associated with lower risk of unplanned healthcare encounters (13).

Beyond wearable fitness trackers, digital consumer devices and smartphone apps have developed increasingly sophisticated health monitoring capabilities with many devices achieving equivalent accuracy and performance to research-grade systems. Collectively termed “health tech,” these devices are capable of producing vast amounts of individual health data on activity, physiology, behavior and anthropometry for use in digital health applications. While performance status remains an important component of lung cancer care, new digital technologies capable of continuously monitoring patients for toxicity, organ dysfunction and disease progression will be important tools for defining the next generation of prognostic biomarkers. In this review, we provide an overview of digital health technologies that could be used across the lung cancer continuum to improve patient risk stratification, enhance operative planning and tailor postoperative disease management.

## Overview of digital health devices for performance status assessment

Digital health wearables and mobile health applications are increasingly used across medical specialties to complement traditional performance status assessments with objective physiological and activity metrics, at variable levels of technology maturity (Table 1). These devices offer continuous monitoring and real-time data collection, providing a more comprehensive and dynamic picture of a patient's functional capacity, especially in chronic illness. By integrating these technologies into clinical practice, oncologists, surgeons and anesthesiologists can gain valuable insights into patients' physical activity, sleep patterns, cardiovascular and pulmonary function, and overall health status. AI and ML algorithms can analyze the continuous stream of data from wearables, identifying patterns and predicting potential complications, which allows for more personalized and timely interventions, potentially improving patient outcomes, particularly during preoperative planning and postoperative recovery.

**Table 1:** Digital and wearable alternative assessments of performance status.

	Modality	Measurements	Example devices	NASA TRL scale^a^
Activity	Exercise Monitors	Physical Activity Intensity, Dose and Fragmentation, Mean Distance Walked	Fitbit Charge HR, ActiGraph Link	8
	Sleep Monitors	Sleep Efficiency, Dose, Awakenings and Latency	Fitbit Charge HR, ActiGraph Link	8
Physiology (Cardiovascular)	Heart rate monitors	Heart rate minimum, maximum, recovery, variability	Polar H10, Fitbit Charge HR	9
	Electro-cardiography	RR interval, ST segment, ST/HR index, chronotropic index, PVC burden	Apple Watch Series 4+, KardiaMobile	9
	Blood pressure	SBP, DBP	Omron HeartGuide, InBodyWatch	9
	Echo-cardiography	LVEF (rest and stress), LVESD, LVEDD	Butterfly iQ, Philips Lumify	7
	Phono-cardiography and phono-pulmonography	Heart sounds, lung sounds, murmur classification	Eko CORE	6
Physiology (Pulmonary)	Photo-plethysmography	SpO2, PVI	Fitbit Sense, Wellue O2Ring, Apple Watch Series 6	8
	Respiratory rate	Respiratory rate (rest and stress), Apnea hypopnea index (sleep)	Fitbit Sense	8
	Spirometry	FEV1, FVC, PEFR	GoSpiro, Aluna	7
	Bioreactance and bioimpedance	Z (impedance)	Zoll uCor	6
Anthropometry	Body composition analysis	Lean body mass	InBody BAND	8
	Ultrasonography	Muscle thickness	Butterfly iQ, Philips Lumify	7

^a^ The NASA Technology Readiness Level (TRL) scale is a systematic metric that assesses the maturity level of a particular technology. It ranges from 1 to 9, with each level representing a stage in the development process from basic research to deployment. In the context of digital health devices for performance status assessment in lung cancer patients, the TRL scale helps determine the readiness of various wearable technologies for clinical use. Technologies at higher TRL levels (8–9) are typically more mature and ready for widespread clinical implementation, whereas those at lower levels (5–7) may still be in the prototype or validation stages, requiring further development and testing before being fully integrated into clinical practice.

**Figure 1:**
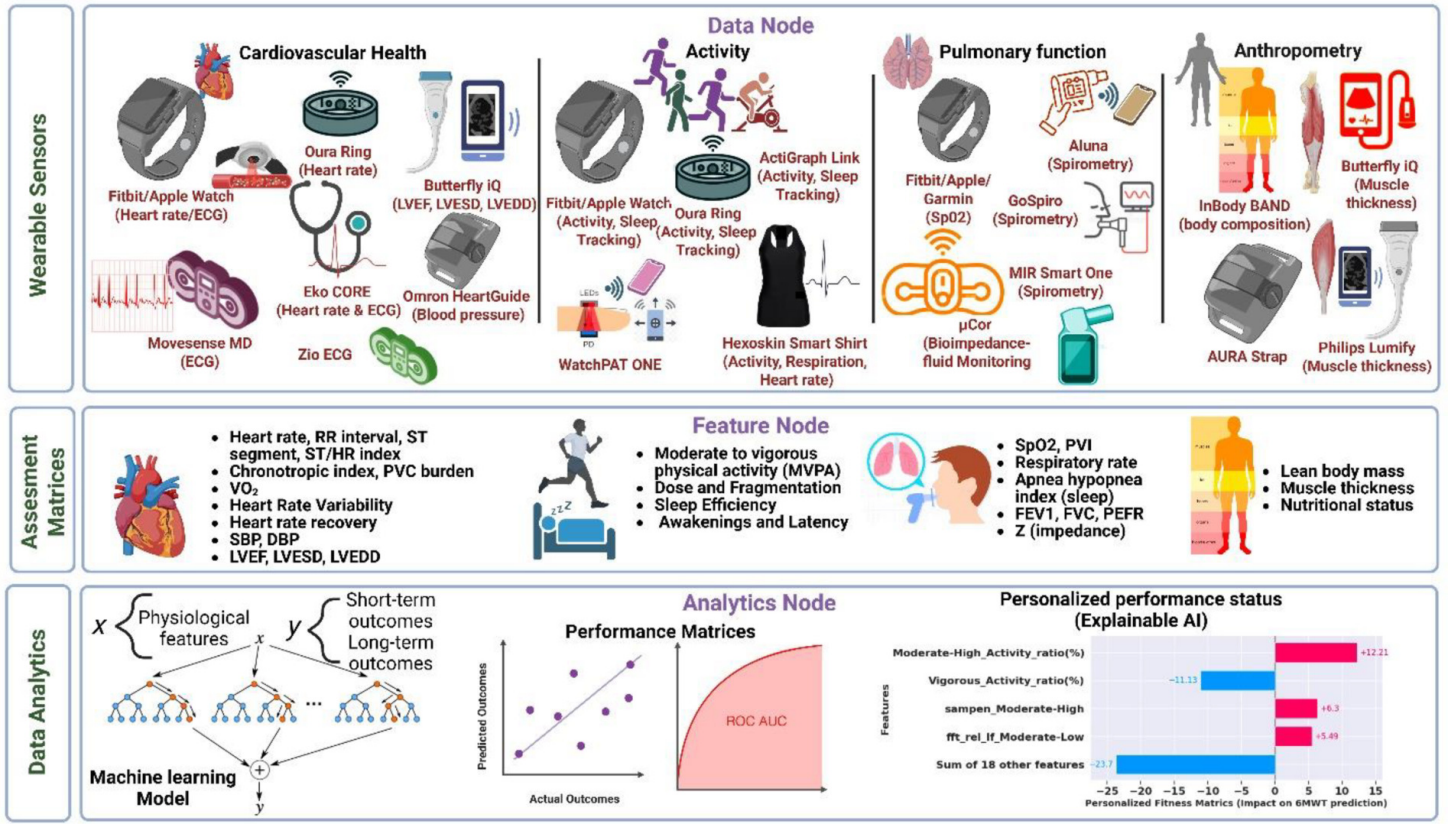
Comprehensive overview for predicting performance outcomes using wearable technologies and AI techniques in lung cancer continuum. Data Node: Heart rate, activity, cardiopulmonary, and body composition data from wearables worn by lung cancer patients. Feature Node: Extraction of physiological and activity features from the wearable data. Data analytics: Application of machine learning algorithms to predict cardiopulmonary fitness and personalized fitness explanation.

Digital health technologies for performance status assessment include a wide range of consumer-grade wearable devices, such as fitness trackers and heart rate monitors, to portable medical devices, such as handheld ultrasound devices and digital stethoscopes (Figure 1). Activity measurements from wearable devices can track physical activity intensity, duration, and patterns in at-home daily living. Unlike subjective exercise tolerance surveys, wearable activity metrics help objectively assess a patient's overall activity levels, which are crucial for understanding their functional capacity. Physiological metrics obtained from wearable devices can provide detailed insights into cardiovascular and pulmonary function, which are critical for assessing a patient's health status and response to treatment. Additionally, anthropometry measurements are validated indicators of nutritional status, physical fitness, and frailty, important considerations for surgical risk stratification and disease prognosis (14).

## Potential applications of digital health devices across the lung cancer continuum

### Diagnosis and initial assessment

Digital wearable devices can play a central role in the diagnosis and initial assessment phase of lung cancer care. Several of these devices are capable of continuous monitoring of various physiological parameters and real-time data collection, providing valuable insights into a patient's baseline functional capacity and predict progression-free survival (15). Continuous monitoring of activity levels, heart and respiratory rate, and sleep patterns can identify early signs of functional decline, which is particularly important for detecting subtle changes that may indicate disease progression (16). Objective measurements of physical activity, cardiovascular health, and pulmonary function from wearables provide a comprehensive baseline assessment essential for future comparisons and evaluating the effectiveness of interventions. Additionally, data from wearable devices can aid in stratifying patients based on their functional capacity, allowing clinicians to tailor treatment plans to individual needs. A recent study by Ito and colleagues, found that wearable-measured mean distance walked accurately classified ECOG PS of 2 or higher, and was associated with better 6-month survival status (17). Conversely, patients with lower activity levels and poorer cardiovascular metrics may require more intensive monitoring and support, particularly in the perioperative period. Furthermore, wearable devices can monitor vital signs and physiological parameters, aiding in the assessment of surgical and treatment risks. Patients with abnormal breathing patterns, irregular heart rate variability or poor sleep quality, for instance, may be at higher risk for respiratory complications, and this information is critical for preoperative and treatment planning (18, 19).

### Treatment and monitoring

During treatment, wearable devices can be instrumental in monitoring patient response and managing adverse effects, providing continuous data to inform treatment adjustments and enhance patient care. Regular tracking of physical activity, heart rate, and respiratory parameters helps assess how well patients are responding to treatments such as chemotherapy or radiation therapy, with decreases in activity levels or changes in physiological metrics potentially indicating adverse reactions or disease progression. Wearable devices can also detect early signs of treatment-related side effects, such as cardiotoxicity or respiratory distress, allowing for timely interventions to mitigate these side effects and improve patient safety and comfort. Ohri and colleagues studied wearable-measured activity in 50 subjects with NSCLS undergoing concurrent chemoradiation therapy, and found that inactive subjects were more likely to be hospitalized (HR 5.6) and less likely to complete radiation therapy without delay (15). Additionally, monitoring devices can track patient adherence to prescribed exercise regimens, prehabilitation, or physical therapy, ensuring that patients follow recommended activity levels and helping healthcare providers support patients in maintaining their functional capacity during treatment (20). Furthermore, wearable technology enables remote monitoring of patients, reducing the need for frequent hospital visits and providing continuous care, which is particularly beneficial for patients with mobility issues or those living in remote areas. Wearable data-driven physiological biomarkers enhance the evaluation of health status and treatment progress, leading to better patient outcomes and quality of life during treatment.

### Surgical resection

Surgical resection remains the mainstay of treatment for all patients with stage I–IIIA NSCLC, and an accepted treatment modality in a minority of patients with advanced or metastatic disease. Patients with early NSCLC commonly have favorable prognosis following resection. However, as advanced surgical techniques have improved immediate perioperative outcomes and extended surgical resection to more complicated patients, there are more patients facing risk of long-term disability, chronic respiratory insufficiency or ventilator-dependence. Pulmonary function testing with laboratory-measured spirometry and diffusion capacity of carbon monoxide (DLCO) is the minimum testing performed prior to substantial lung resection surgery, with more advanced testing such as lung scintigraphy or CPET reserved for patients with lower baseline forced expiratory volumes or diffusing capacity. Combining these preoperative measurements with the surgical resection plans has traditionally been performed to predict postoperative (ppo-) pulmonary function (ppo-FEV1 and ppo-DLCO), but this has been shown to significantly underestimate actual postoperative measurements, limiting their utility for surgical planning (21, 22).

Preoperative wearable data can be pivotal in guiding surgical and anesthetic planning for lung cancer resections. For instance, in place of formal cardiopulmonary exercise testing, estimated VO2max or anaerobic threshold obtained from preoperative physical activity monitoring can help determine the risk of postoperative pulmonary complications and long-term sequelae. Additionally, preoperative monitoring of pulse oximetry during sleep can identify patients with undiagnosed obstructive sleep apnea (OSA), allowing for adjusted narcotic dosing or multimodal analgesia to minimize respiratory complications. Home spirometry, including measurements of FEV1 and FVC, can be used to predict the patient's ability to tolerate intraoperative one-lung ventilation or the likelihood of successful extubation. Integration of this digital data into the surgical and anesthetic plan can enhance patient safety by personalizing care with objective physiological data.

### Survivorship

For lung cancer survivors, wearable medical technologies are vital in ongoing health monitoring, particularly concerning physical activity levels, respiratory rate, and heart rate variability. These devices deliver objective data that help assess survivors' functional capacity and predict long-term outcomes. Continuous monitoring of respiratory rate and heart rate variability provides crucial information on cardiopulmonary function, aiding in the management of long-term health. Wearables also enable pulmonary rehabilitation to be performed at home and without direct supervision. In a study of 64 patients with NSCLC, Ji et al. found that a 12-week mobile health-based pulmonary rehabilitation program significantly improved 6-minute walk distance, dyspnea, and quality of life (23). Wearable technologies thus support survivors in maintaining health and preventing complications, enhancing overall quality of life.

**Figure 2:**
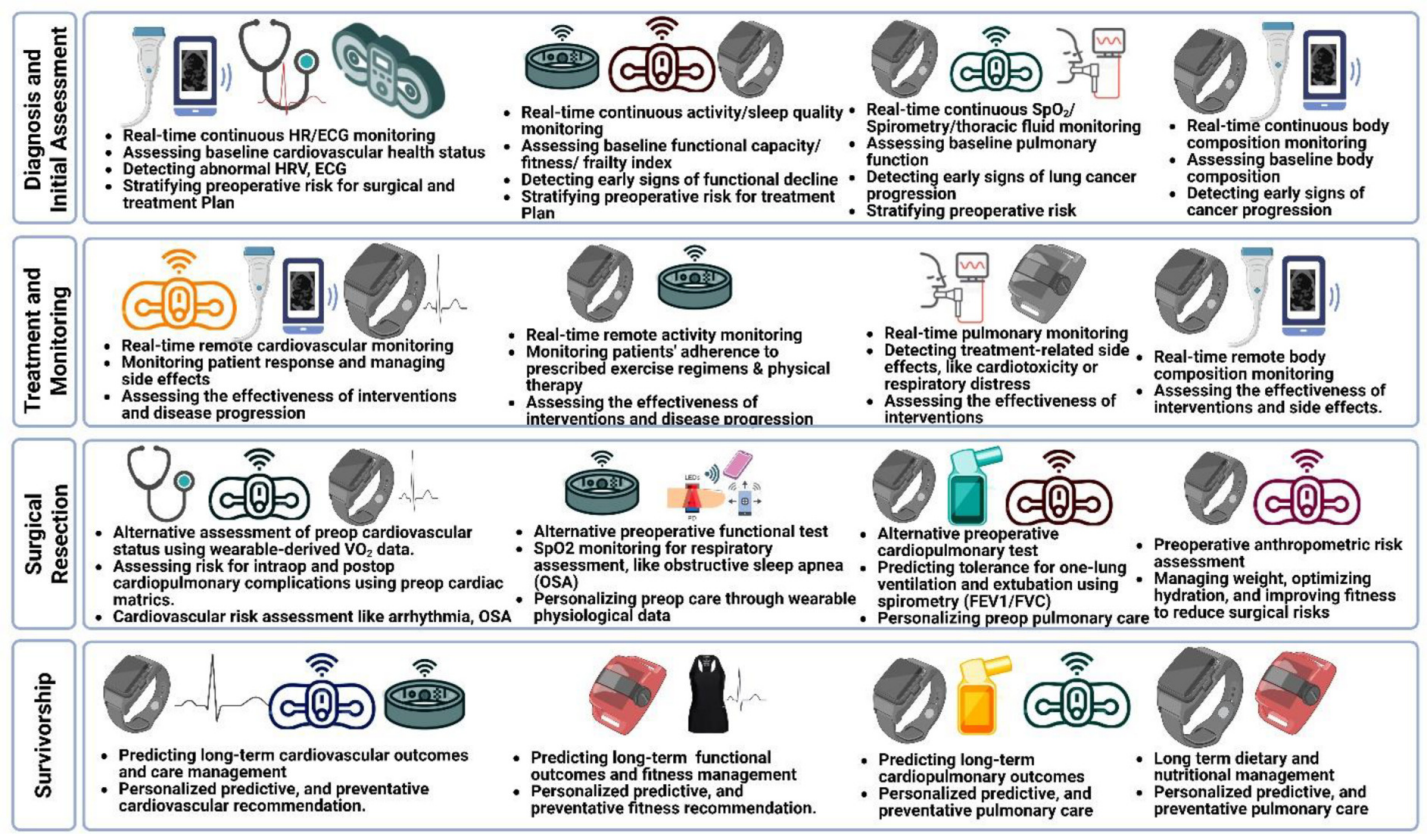
Potential application of digital health devices across the lung cancer continuum. Diagnosis and Initial Assessment: Heart rate, activity, cardiopulmonary, and body composition data from wearables for early diagnostics and preoperative risk stratification. Treatment and Monitoring: Real-time wearable data for monitoring patient response and disease progression. Surgical Resection: Alternative physiological monitoring for assessing risk for intraoperative and postoperative cardiopulmonary complications. Survivorship: Long-term personalized predictive, and preventative cardiopulmonary care.

The application of digital health technologies across the lung cancer continuum, from diagnosis and initial assessment through survivorship, is summarized in Figure 2.

## Discussion and future directions

AI-integrated digital health technologies have progressed far beyond the traditional performance status assessment and have the potential to transform lung cancer management and thoracic surgery. Wearables can provide objective data on patients' physical activity levels, pulmonary function, and cardiopulmonary health, which can be used to assess disease progression, predict outcomes, optimize surgical planning, and identify complications early. Wearables can provide efficient and user-friendly estimates of accepted gold-standard measures, like FEV1 and VO2max; data streams like these would be comparable to current ones, so existing clinical infrastructure, workflows, and roles should be able to accommodate wearable-acquired data without significant change. Novel or nonstandard measures collected by wearables can provide new insights into physiology, especially in the real world; with further clinical validation and the help of ML algorithms to analyze these data streams, digestible and actionable information can be provided directly to clinicians. In the age of electronic health records and AI, clinician decision support for wearables-acquired data should be relatively straightforward to implement, as similar pathways already exist for standard data like clinical notes, images, and laboratory values. By integrating these existing technologies into clinical practice, healthcare providers can readily enhance the quality of care for lung cancer patients, likely improving both short-term and long-term outcomes.

Increased consumer access to these novel personal wearable health technologies has opened new frontiers in data collection and model training (24, 25). Large-scale studies, like the Apple Heart Study, are able to enroll an astounding number of participants by taking advantage of increasing ubiquity of wearables (26). A unique advantage of these technologies is the ability for investigators to automate the enrollment of patients on personal devices (27). Additionally, while concerns about technological literacy and barriers to entry for wearables exist (28, 29), traditionally underrepresented populations, including those with rare disease and from minority health groups, may have increased research visibility via ease of data collection as digital health technologies become more accessible (25).

Next-generation digital health technologies promise to build on these advances in thoracic oncology care by monitoring multiple physiologic measures simultaneously and continuously with low-profile extended-use devices. These include wearable patches to detect metabolites, electrolytes, hormones, and nutrients (30–34); wearable user-independent ultrasound that can record multi-parameter echocardiography, cerebral and peripheral blood dynamics, and various physiologic waveforms (35–37); and motion-robust wearable neuroimaging integrated with EEG, ECG, blood pressure, and more (38, 39). These experimental (lower TRL, non-commercial) devices have the potential to provide dynamic, holistic pictures of patients across their care continuum without additional patient effort or discomfort. They can also continuously integrate readouts across organ systems to support even finer risk stratification for patient-centered outcomes, like cognitive function or pain, and to facilitate real-time and ongoing decision-making, such as for electrolyte correction, nutritional optimization, or treatment efficacy. Rapid maturation and validation of these novel technologies requires prioritization by clinical partners. Overall, the application of current and emerging digital health technologies can promote precision lung cancer care and elevate health and longevity, while empowering patients to participate in their care without undue burden.
